# Hop (*Humulus lupulus* L.) Phytochemical Profiles as a Function of Growth Region by HPLC and GC-MS Analysis

**DOI:** 10.1021/acsomega.5c07649

**Published:** 2026-01-16

**Authors:** Celina Paoletta, Christopher Balog, Andrew Higgs, Dmitry Liskin, Abigail Brehm, Kevin Kingsbury, Ronald A. Quinlan

**Affiliations:** † 6013 Christopher Newport University, 1 Avenue of the Arts, Newport News, Virginia 23606-2998, United States; ‡ Tradition Brewing Company, 700 Thimble Shoals, Newport News, Virginia 23606, United States

## Abstract

There is growing interest from brewers, hop growers and consumers about the regionality of hops as affected by growth region. The present work aims to characterize the pelletized hop (*Humulus lupulus* L.) cultivar Cascade from two regions in the United States; Yakima, Washington and Benton County, Minnesota. Analyses were performed using high pressure liquid chromatography with diode array detection (HPLC-DAD) and headspace gas chromatography–mass spectrometry (HS-GC-MS). Materials were obtained from commercial sources, Yakima Chief Hops and Mighty Axe Hops. While the phytochemical profiles were similar, as expected for a single cultivar, differences in the volatile and nonvolatile content ratios were observed. The results of this study support the increasing evidence of hop terroir and highlight the need for continued studies into the effects that local growth environments can have on the analytical profile of hops. These results will be of particular interest to the discerning brewer that is looking for the ability to craft exceptional flavor profiles for their finished products.

## Graphical abstract

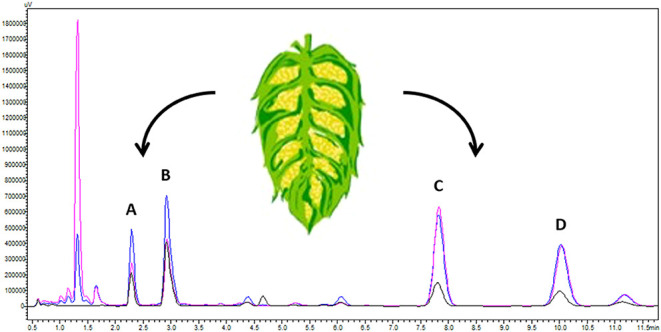

## Introduction

The importance of hops (*Humulus lupulus* L.) in brewing is well-known.^1,2^ They are primarily used as a bittering agent, contributor to aroma, flavor and as a preservative due to their antimicrobial properties.^3^ Other metabolites found in hops can contribute to other qualities such as foam stability, color, and mouthfeel.^4^ Additionally, various health-promoting components in beer can be attributed to hops, such as amino acids, bitter acids, carbohydrates, flavonoid compounds, and vitamins.^3^ The α- and β-acids are considered to be the predominately bittering compounds that balance the sweetness of the wort and while the β-acids (lupulone, colupulone, and adlupulone) contribute to the overall bitterness, the α-acids (humulone, cohumulone, and adhumulone) are generally considered to be the precursors of the bitter compounds as they isomerize during the brewing process.^1,4–6^ The two general functions that many brewers consider when choosing a hop varietal are bittering and aroma, with higher α-acid content hops being chosen for bittering and lower α-acid content hops being chosen for aroma, however, there are hop varietals that can serve as dual purpose hops.^7^

During the brewing process, hops are typically added as the mashed malted barley or malt extract is boiled in water.^2,7^ Bittering hops are added closer to the beginning of the boiling stage whereas aroma hops are added toward the end of the boiling stage because the essential oils in hops that impart flavor and aroma quickly evaporate. As this mixture boils, the modestly bitter α-acids undergo thermal isomerization and form extremely bitter *cis*- and *trans*-iso-α-acids. The more soluble and stable iso-α-acids have the strongest influence on the bitter flavor of beer. During the boiling process, the β-acids oxidize to produce a bitter flavor. While these oxidation products impart a harsher bitter flavor, the extent is still marginal compared to α-acids because the oxidation products are relatively insoluble. However, dry-hopping is a brewing technique where hops are added after the wort is cooled, typically during fermentation or aging to extract more of the favorable volatile and nonvolatile compounds while minimizing the extraction of the bitter acids.^7,8^ The bitterness of a beer mainly depends on the concentration of α-acids contained in the hops, the amount of hops used, and the length of time the hops are boiled–resulting in the formation of iso-α-acids.^1,4–6,9,10^

There are over 100 variety of hops and each variety has their own character, so brewers are able to alter the flavor of a beer depending on the cultivar of hops selected. Breeding programs aim to create new cultivars of hops to improve the flavor and aroma profile of hops for brewing, which may involve breeding hops to increase the production of the α-acid bittering compounds, while improving yield and disease resistance.^7^ A favorite cultivar of hops that many are familiar with is Cascade. The profile of Cascade is described as strong, spicy, floral, with a citrus (i.e., grapefruit) aroma. Another common cultivar is Mosaic, whose profile includes berry, citrus, stone fruit, and tropical notes. Hops with a higher α-acid content, like Mosaic, typically contribute more to bitterness, but tend to impart a less refined flavor and aroma to a brew. Hops with a lower α-acid content, like Cascade, typically contribute a desirable flavor and aroma to a beer.^7^ Thus, the chemical composition of hops will vary depending on the cultivar.

Hops are the cone-shaped flowers of the *H. lupulus* plant. The hop plant is native to the regions with temperate climates in North America, Europe, and Asia, typically near the 40–50° latitudes, specifically Yakima Valley in Washington, the Hallertau region in Germany, and New Zealand, which are among some of the places that meet the criteria for thriving hop plants and are well-known for their production of hops.^8,11^ The bines of a hop plant are vine-like structures that wrap around any object within reach as it grows toward light and can extend 20 feet or more. The hop plant is dioecious—has separate male and female plants—and the flowers of the female plant are much larger than the male plant. The female hop plant is used for brewing because the flowers or strobili are much larger in size and contain more essential oils and resins that are utilized for their bittering and aroma qualities.^11^ The essential oils and resins are produced within the lupulin glands of the hop flower. A cross section of a hop cone with the labeled anatomy is provided in Figure 1. The total resins found in the lupulin glands consist of soft resins and hard resins. The bitter acids are present within the soft resins and the oxidized bitter acids as well as xanthohumol–a prenylated flavonoid with antioxidant and anti-inflammatory properties–are present within the hard resins.^12^

**Figure 1:**
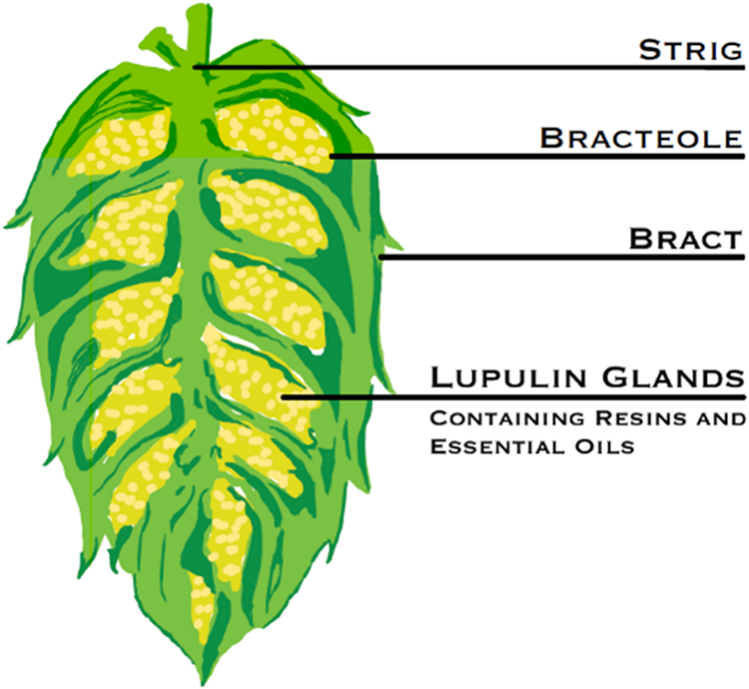
A cross section of a hop with the labeled anatomy.

The essential oils contain more than 250 chemical compounds, including the terpene and terpenoid classes of organic compounds that are responsible for contributing to the flavor and aroma of beer.^8,11^ Humulene, caryophyllene, farnescene, and myrcene are prevalent in hops with each compound possessing their own distinct flavor and aroma characteristics. Some of the modified versions of these compounds consists of geraniol, linalool, pinene, and nerol that also produce various flavors and aromas that are present in hops as well. Each hop cultivar possesses unique flavors and aromas due to the varying chemical composition of the essential oils and amount of bitter acids.^11^

While it is known that the α- and β-acid content differs depending on the cultivar, it has been shown more recently that acid profiles and aroma profiles of a specific cultivar of hops can vary even further due to differences in the environment the hops are grown in.^10,13–18^ The effect that changes in the environment–soil, climate, and topography–has on acid profiles, potentially resulting in an alteration to the flavor and tasting experience, is known as terroir.^13,19,20^ However, while studies investigating the extent that terroir has on altering the acid profile and aroma profile of individual cultivars of hops are limited, early work does indicate that there are some regionality differences of fresh, whole hops.^13,20^ Also, recent efforts have shown that packaging can influence aroma and flavor compounds originating from hops.^21,22^ Therefore, the primary goal of this effort is to develop a deeper understanding of the influence terroir has on hop profiles of the same cultivar, but specifically of commercially available hop pellets that local craft breweries and home brewers would utilize. High performance liquid chromatography with diode array detection (HPLC-DAD) was utilized to examine nonvolatile extracts, while headspace gas chromatography with mass spectrometry (HS-GC-MS) was utilized to compare and contrast the volatile components of the hops. Due to potential differences as a result of blending during packaging or due to regionality, obtaining the phytochemical profiles of the hops is important for brewers with regard to quality control and flavor profiles, especially when trying to create new tasting experiences.

## Materials and Methods

The liquid chromatographic system used was a Shimadzu Nexera 40 Series HPLC using a Restek Raptor AR C18 (5 μm, 150 mm × 3.0 mm) column and a Shimadzu SPD-M40 photodiode array detector scanning from 200 to 800 nm. The mobile phase consisted of an aqueous phase (A) made up of 18 MΩ water (in-house Milli), HPLC grade methanol (Sigma-Aldrich), HPLC grade phosphoric acid (85%, Fisher Scientific), and HPLC grade triethylamine (Fisher Scientific) in a 300 mL/700 mL/19.6 g/15.1 g ratio. The organic phase (B) was pure methanol. Separation was achieved with gradient elution, with B conc. 0% (0 min) to 35% (10 min). The B concentration was held at 35% for the remainder of the full 30 min run time to ensure elution of the compounds. Blank samples were added between samples to confirm no carryover between samples. To prepare hop pellets for analysis, approximately 10 g were pulverized until homogeneous. Then 2.5 g of homogenized hop pellets were placed into a 125 mL Erlenmeyer flask and 25 mL of toluene (HPLC-grade, Fisher Scientific) added. The mixture was shaken for 30 min. The toluene extract was centrifuged, and rotary evaporation was used to concentrate 5 mL of the supernatant. The residue was dissolved in a 25 mL addition of methanol (HPLC-grade, Fisher Scientific). Then, 2 mL of the hop extract was filtered through a 0.45-μm nylon filter into an HPLC sample vial. LabSolutions LCsolutions (version 5.97 SP1) software was used to run the sequences and automate the integration of chromatograms.

The gas chromatographic system used as a Shimadzu GCMS-QP2020 NX with a PAL AOC-6000 plus autosampler, equipped with the headspace tool. A Stabilwax (crossband/carbowax/poly­(ethylene glycol)) polar column (60 m × 0.25 mm ID × 0.5 μm film thickness, Restek) was used for separation, the split ratio was 1:10, and the carrier gas was helium at a linear velocity of 40 cm/s. The temperature program started at 35 °C and was held for 1 min, then the temperature increased to 60 °C at a rate of 30 °C min^–1^, and then increased to 200 °C at a rate of 8 °C min^–1^ and was held for 5.5 min, resulting in a total run time of 24.83 min. The mass selective detector was set to operate in electron impact ionization mode at 70 eV, the scan range was 35–300 *m*/*z* with a scan speed of 3333 u/s, and the start time of the MS was 2.60 min with an end time of 24.83 min. The ion source temperature was 250 °C, the interface temperature was 200 °C, and the solvent cut time was 1.95 min. Hop samples were prepared by simple distillation utilizing approximately 15 g of pellets. The pellets were placed in 500 mL round-bottom flasks with 150 mL of 18 MΩ water and were set to boil for 60 min to simulate the boiling stage of the brewing process. Approximately 15 mL of distillate was collected for each simple distillation and was stored in respective 30 mL GC sample vials. Samples were stored for approximately 12 h in the refrigerator before removing approximately 12 mL of the distillate to separate 20 mL, amber HS sample vials.

The hop samples consisted of Cascade hops grown from Yakima, Washington (Yakima Chief Hops) and Cascade hops grown in Minnesota (Mighty Axe Hops). Standards included the ICE-4 (American Society of Brewing Chemists) and Cascade Hop Oil (Aromatics International). The ICE-4 standard was prepared for HPLC-DAD analysis by dissolving 0.1 g of the standard extract in 100 mL of methanol (HPLC-grade, Fisher Scientific). Then, 2 mL of the dissolved hop extract was filtered through a 0.45 μm nylon filter into an amber HPLC sample vial. The Cascade essential oil was used as purchased by placing five (5) drops in the amber HS vial before sealing.

## Results and Discussion

### HPLC-DAD Analysis

The ASBC ICE-4 standard, Yakima Cascade hops, and Minnesota Cascade hops were analyzed by HPLC-DAD to produce the respective chromatograms (Figure 2). Enlarged views of each peak grouping are provided in the Supporting Information (S1–S4). Peak assignments were based on relative percent composition ratios and expectations of elution order as described in ASBC method Hops-14, as well as previous literature.^23^ The order of elution with the respective retention times for the α- and β-acids present in the ICE-4 standard are summarized in Table 1. As is shown in Figure 2, the overlay of the chromatograms is almost exact. Interestingly, the percent composition of cohumulone in the Washington hops was greater than that of the Minnesota hops (+7.704%), and for adhumulone + humulone (+14.053%). The percent composition of colupulone in the Washington hops was less than the Minnesota hops (−2.270%) and the composition for adlupulone + lupulone was almost the same, with Washington hops having a slightly larger composition (+0.411%). The Washington hops had a total α-acid composition of 57.323% and a total β-acid composition of 41.571%, while the Minnesota hops had a total α-acid composition of 35.567% and a total β-acid composition of 43.431%. That the peak composition parameters do not add to 100% is caused by the 4–5 small peaks found between 4.0 and 6.5 min (Figure 3). While these numbers are not exact, a more detailed analysis and quantification would need to be performed such as presented by others,^23^ what is interesting to the discussion is the relative values of α- to β-acid composition. Carbone et al.^18^ recently described differences in Cascade hops grown in two Italian regions (Latium and Tuscany). While differences in bitter acid content were indicated, the analysis focused on the distinct sensory panel and the gas chromatography-olfactometry analysis. The emphasis for the bittering acids was based on a ratio of cohumulone to total α-acids present and by percent weight on dry basis. Perhaps of interest, for an additional study, is the reported values of 18.28 and 19.45 for the cohumulone ratio are very similar to the value of 18.96 reported for the Washington hops. Their findings suggested that the rurality of the growth area plays a role in the differences. Rodolfi et al.^17^ also investigated Cascade cultivars from samples in the United States, Germany, Slovenia, and Italy. Of particular interest, the two regions selected from the United States were Oregon and Michigan. Our analysis agrees with their study in that the Pacific Northwest hops had higher quantities of α-acids than the Midwest hops. However, the differences for β-acids that we observed do not appear to be as significant. Forster and Gahr^24^ compared Cascade from Yakima, Washington and Hallertau, Germany. Their results for the α-acids showed a dependence upon the year and only focused on total acid content and cohumulone ratio as well. While the effect of each α- to β-acid homologue has yet to be determined, it is known that bitterness derived from rho-iso-α acids (RIAA), tetrahydro-iso-α acids (TIAA), and hexahydro-iso-α acids (HIAA) can be influenced by the matrix and can be very different.^1,25,26^

**Figure 2:**
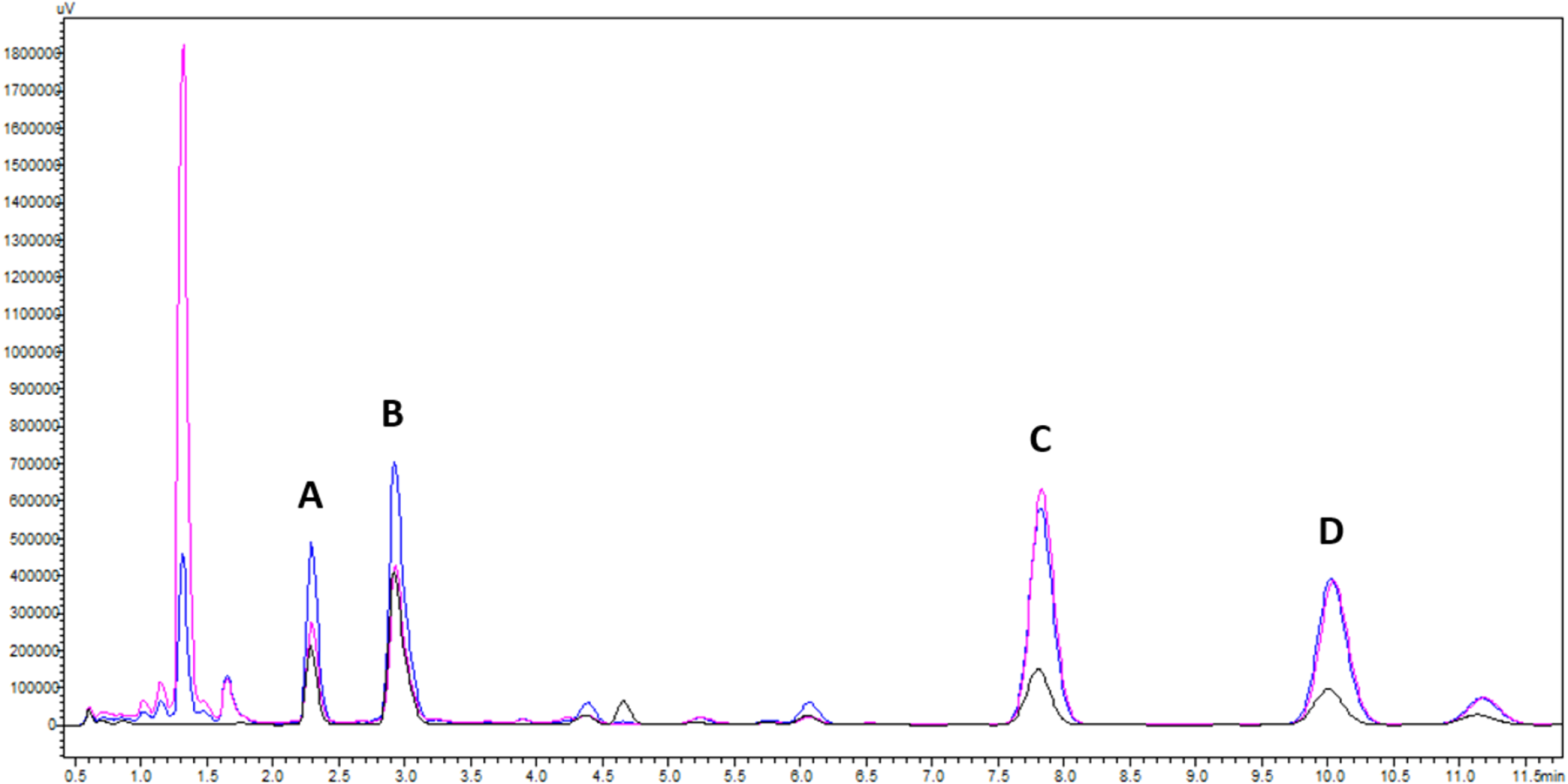
Overlayed HPLC-DAD chromatograms of the ASBC ICE-4 standard (black), Washington hops (blue), and Minnesota hops (pink). The respective peaks were identified as cohumulone (A), adhumulone + humulone (B), colupulone (C), and adlupulone + lupulone (D).

**Figure 3:**
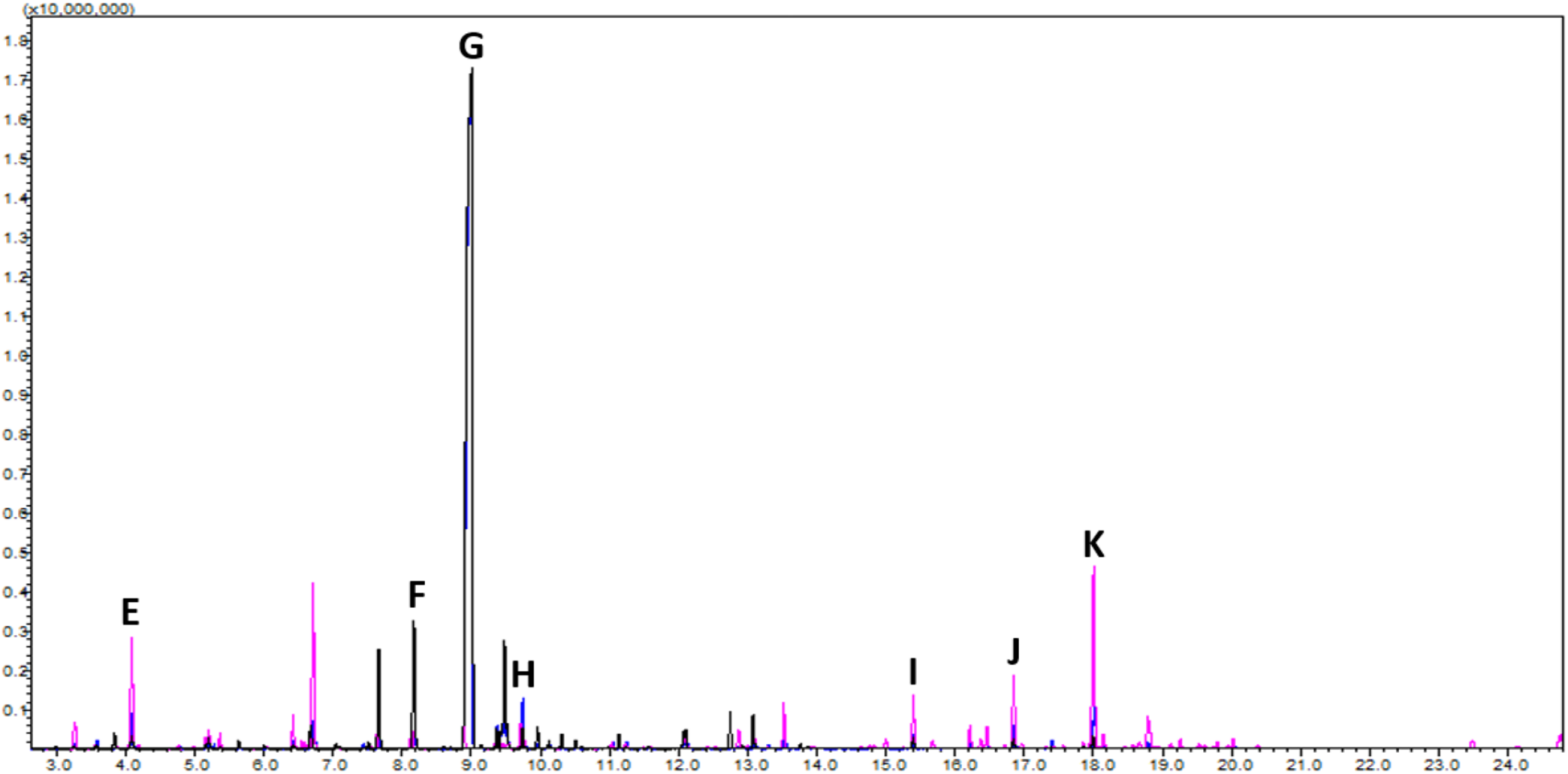
HS-GC-MS chromatogram comparison of Aromatics International Cascade Hop Oil (black), Washington hop distillate (blue), and Minnesota hop distillate (pink). The respective peaks identified were (E) 1-(5-(6-Chlorobenzo­[*d*]­thiazol-2-yl)­furan-2-yl)­ethyl acetate, (F) β-pinene, (G) β-myrcene, (H) d-limonene, (I) linalool, (J) caryophyllene, and (K) humulene (α-caryophyllene).

**Table 1:** Retention Time and Percent Composition of the Primary Components Based on the International Calibration Extract 4 (ICE-4) as Purchased from the American Society of Brewing Chemists (ASBC)^a^

	ASBC ICE-4		Washington		Minnesota	
acid grouping (peak)	ret. time (min)	comp. (%)	ret. time (min)	comp. (%)	ret. time (min)	comp. (%)
cohumulone (A)	2.288	10.98	2.294	18.96	2.295	11.26
adhumulone + humulone (B)	2.920	31.60	2.925	38.36	2.931	24.31
colupulone (C)	7.804	13.02	7.821	22.38	7.829	24.65
adlupulone + lupulone (D)	10.003	13.52	10.024	19.19	10.041	18.78

a The percent compositions were determined using Shimadzu’s LabSolutions software and representative of the four main peaks.

### GC–MS Analysis

An overlay of representative chromatograms are provided in Figure 3. Enlarged chromatographs are provided in the Supporting Information (S5–S11). The Aromatics International Cascade oil chromatograms produced a total of 24 peaks that were automatically integrated by the LabSolutions GCMSsolutions software (version 4.54). Of those 24, 23 compounds were identified by the software using the standard NIST library (version 2020 by Wiley). Of those 23 identified, only 11 also appeared on the qualification report provided by Aromatics International, which indicated there should be 32 compounds present, consisting of monoterpenes, sesquiterpenes, esters, diterpenes, monoterpenols, and ketones. The Washington distillate produced chromatograms with 19 total peaks and 18 that were identified. Of the 18 identified, only 7 corresponded with the Aromatics International sample. The Minnesota distillate produced chromatograms with 31 peaks and 29 successfully identified. Of the 29, only 11 corresponded with the Aromatics International sample. The full chromatogram data with available odor descriptors are provided in Supporting Information. There were seven (7) compounds that were successfully identified in all three essential oil samples (Table 2) and zoomed-in overlays of these regions of the chromatograms are also provided in the Supporting Information. The similarity index (SI) value provided by the GC-MS software for the qualification of peak E ranged from 88 to 90 across the three chromatograms, while the SI values for the qualification of peaks F–K ranged from 95 to 97 across the three chromatograms. It should be noted that 1-(5-(6-Chlorobenzo­[*d*]­thiazol-2-yl)­furan-2-yl)­ethyl acetate is not a compound expected to be identified in a hop essential oil analysis, but since it was identified in all three samples, it is included in the discussion. Full peak identifications are provided in the Supporting Information (ST.1–ST.3), but since confident comparisons could not be made across all samples the discussion of these differences needs further analysis and is left for continuing efforts.

**Table 2:** Volatile Compounds Successfully Identified by GC-MS in both Washington and Minnesota Hops, as well as the Aromatics International’s Standard Sample

compound (peak)	Aromatics International	Washington	Minnesota
1-(5-(6-chlorobenzo[*d*]thiazol-2-yl)furan-2-yl)ethyl acetate (E)	0.46	1.59	10.61
β-pinene (F)	5.44	3.28	2.08
β-myrcene (G)	75.28	77.52	2.16
d-limonene (H)	0.73	2.30	2.04
linalool (I)	0.35	0.58	4.23
β-caryophyllene (J)	0.32	1.10	7.12
humulene (K)	0.46	2.05	19.65

While the percent composition numbers are not quantitative, they serve the purpose of providing relative information for our discussion. The percent composition of 1-(5-(6-Chlorobenzo­[*d*]­thiazol-2-yl)­furan-2-yl)­ethyl acetate in the Minnesota Cascade distillate was greater than the corresponding values obtained for the Aromatics International Cascade Hops Oil and Washington Cascade hops distillate with a difference of 10.15% and 9.02%, respectively. For β-pinene, the determined percent composition was greater in the Aromatic International Cascade Oil compared to the Washington Cascade distillate and the Minnesota Distillate with a difference of 2.16% and 3.36%, respectively. The Washington Cascade distillate had a greater relative percent composition value compared to the Minnesota Cascade distillate with a difference of 1.20%. The percent composition of β-myrcene in the Washington Cascade distillate was greater than the corresponding values obtained for the Aromatics International Cascade Oil and the Minnesota Cascade distillate with a difference of 2.24% and 75.36%, respectively. For d-limonene, the percent composition was greater in the Washington Cascade distillate compared to the composition values obtained for the Aromatics International Cascade Oil and the Minnesota Cascade distillate with a difference of 1.57% and 0.26%, respectively. The percent composition of linalool was greater in the Minnesota Cascade distillate compared to the Aromatics International Cascade Oil and the Washington Cascade distillate with a difference of 3.88% and 3.65%, respectively. For caryophyllene (peak J), the percent composition was greater in the Minnesota Cascade distillate compared to the composition values obtained for the Aromatics International Cascade Oil and the Washington Cascade distillate with a difference of 6.80% and 6.02%, respectively. The percent composition of humulene was greater in the Minnesota Cascade distillate compared to the Aromatics International Cascade Oil and the Washington Cascade distillate with a respective difference of 19.19% and 17.60%, respectively. While the 7 compounds were present in the Cascade oil standard and both distillates, the preliminary data reflects there were differences in the relative composition values for these volatile compounds across the three sample types. The data support similar differences observed in the work of Carbone et al.,^18^ Rodolfi et al.,^17^ and Forster and Gahr.^24^ All 6 of the expected compounds were found in the works of Carbone and Rodolfi. Limonene was not reported by Forster and Gahr. The trends observed, β-pinene, myrcene, and limonene being a larger composition while linalool, caryophyllene, and humulene being a smaller composition in the Pacific Northwest region versus the Midwest region, are the same as observed by Rodolfi et al.^17^ This observation supports the regionality of the Cascade cultivar. Exact comparisons are not possible however as these previous works utilized fresh hop cones and our data utilized pelletized material. Furthermore, the methods of distillate collection should be considered. Carbone et al.^18^ and Rodolfi et al.^17^ utilized a Clevenger apparatus for hydrodistillation while Forster and Gahr^24^ utilized a Buchi Distillation Still. The distillates in this study were a result of only 1 h of boil and an open condenser was utilized for collection. While it was expected that some of the volatile compounds would be lost, it was desired to simulate the brewing environment of our small system, with the ultimate goal being that of a tasting panel. Also of interest is the method of injection for the GC-MS analyses. Recent work by Anderson et al.^27^ reviewed many forms of analyses and there are differences in the headspace (HS) trap, static headspace (HS), and solid-phase microextraction headspace (SPME-HS) methods. Furthermore, the continuing work by the Schug group, specifically Zanella et al.^28^ highlight sensitivity differences in fiber coatings when utilizing the SPME-HS method. These changes can lead to differences in the analyses of compounds and should be taken into consideration by brewers and scientists, depending on their goals. According to earlier work by Van Holle^14^ the aroma profile of a hop is an important asset when determining the regionality, or terroir, influence of the hop. Subsequent efforts by Van Holle furthered the support of terroir-related hop compounds and the traits and characteristics of the resulting beers.^15^

Though the discrepancies of the GC-MS analysis between the Aromatics International Cascade Hops oil and their quality control report may indicate that the method (stationary phase or injection technique) needs to be optimized to detect and determine the other 21 compounds present in the oil, there was adequate separation of peaks and the method is similar to the other literature previously referenced. It may also indicate that the compounds detected in the analysis of the Minnesota Cascade and Washington Cascade, but that were not detected in the analysis of Cascade Hops Oil, may not have been detected due to the volatility, stability, or potential degradation of those compounds that result from differences in extraction methods. As shown in the Supporting Information, there were also identified peaks that had SI values that were below 90 and may cause one to question the accuracy of the qualification. This may indicate that an expanded MS library is necessary. It could also indicate that due to the complexity of the analysis (numerous compounds present in hops oil have similar molecular masses), the use of high mass accuracy instrumentation such as two-dimensional gas chromatography and tandem mass spectrometry is required.^29^

Additionally, work by Lafontaine et al.^30^ has highlighted the importance of harvest time and farm conditions during harvest on the phytochemicals that are responsible for hoppy beer flavor and other characteristics. Recent efforts of Féchir et al.^31^ also identified terroir effects within the Pacific Northwest region as a result of soil characteristics, soil chemistry, and climate. This work builds on the work of De Keukeleire^32^ that found correlations based on organic versus conventional farming as well. Furthermore, since it is known that the human experience of bitterness can vary based on chemosensory organs^33^ and the complexities of interactions between beer components, both known and unknown,^34^ the analytical profile of hop materials may or may not match the sensory expectations of the consumer.

## Conclusions

The present work demonstrated differences in the nonvolatile and volatile composition of hop extracts for the Cascade varietal using HPLC-DAD and HS-GC-MS. The trends observed in this work support previous efforts that have identified regionality, or terroir effects, of hops, though this work highlights comparative ratios of compounds. Additionally, some of the trends are different, highlighting the importance of additional factors, such as soil and farming practices, not just regionality. Comparison of this work to previous efforts highlights the need for additional studies to elucidate the significance of individual factors on the phytochemical profile of hops. Additionally, the relationship between the analytical composition and the sensory experience needs further correlation so that brewers can provide adequate consideration to the providence of hops.

## Supplementary Material



## Notes

Enlarged figures to include the four HPLC-DAD peaks and the seven HS-GC-MS peaks, additionally, the complete HS-GC-MS analyses for each of the three samples; aromatics International Cascade Hop Oil, Washington Distillate and Minnesota Distillate (PDF)

## §

New Belgium Brewing Fort Collins, Colorado 80524–2457, United States
