# Supplementary material for: Hop (Humulus lupulus L.) Phytochemical Profiles as a Function of Growth Region by HPLC and GC-MS Analysis
Source: ACS Omega. 2026 Jan 16;11(4):5241–7. doi: 10.1021/acsomega.5c07649 (PMC12878722; doi:10.1021/acsomega.5c07649)
Supplement: Supplementary file 1 [file ao5c07649_si_001.pdf]

Supporting Information

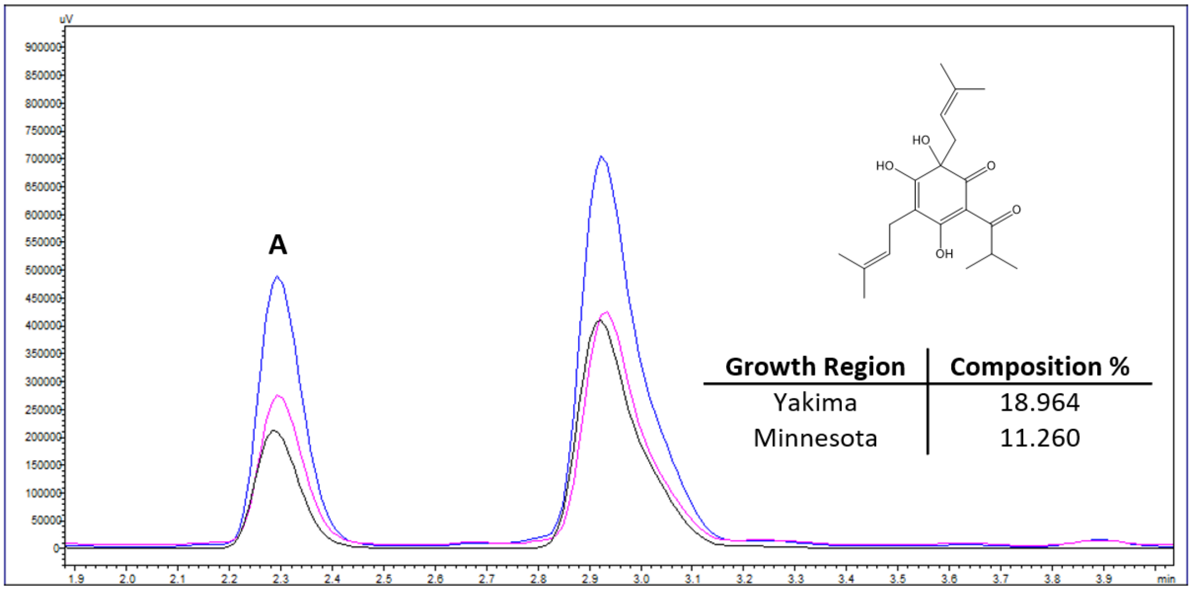

Figure S1. Overlayed HPLC-DAD chromatograms of the ASBC ICE-4 standard (black), Washington hops (blue), and Minnesota hops (pink). The structure of cohumulone is shown as an inset in the upper right with a table corresponding to the composition values in the lower right.

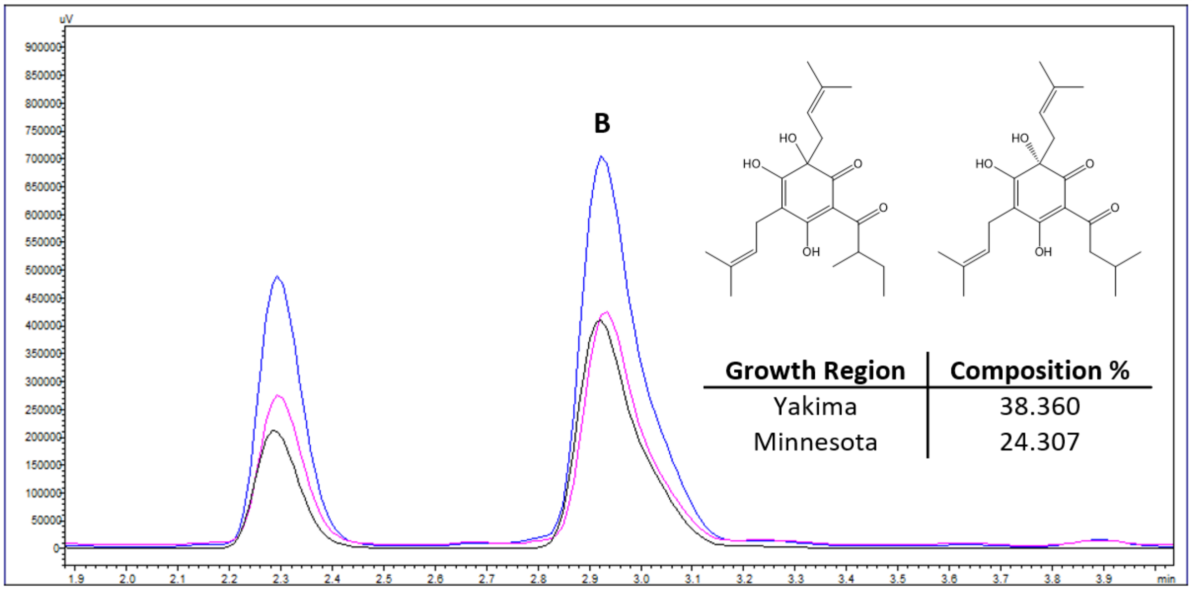

Figure S2. Overlayed HPLC-DAD chromatograms of the ASBC ICE-4 standard (black), Washington hops (blue), and Minnesota hops (pink). The structure of adhumulone (left) and humulone (right) are shown as an inset in the upper right with a table corresponding to the composition values in the lower right.

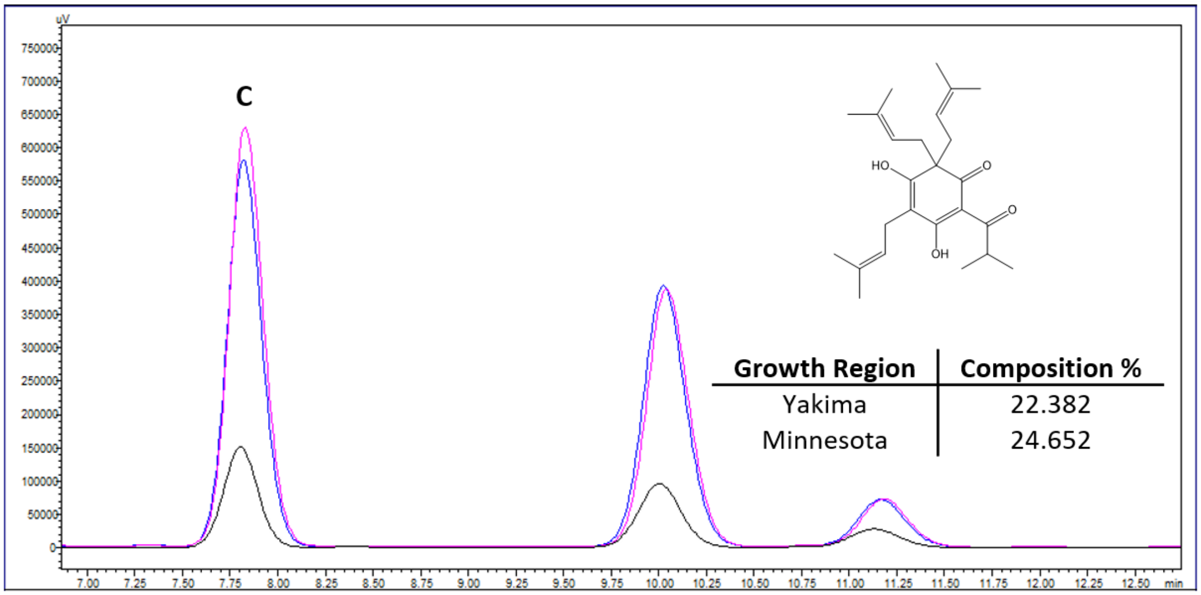

Figure S3. Overlayed HPLC-DAD chromatograms of the ASBC ICE-4 standard (black), Washington hops (blue), and Minnesota hops (pink). The structure of colupulone is shown as an inset in the upper right with a table corresponding to the composition values in the lower right.

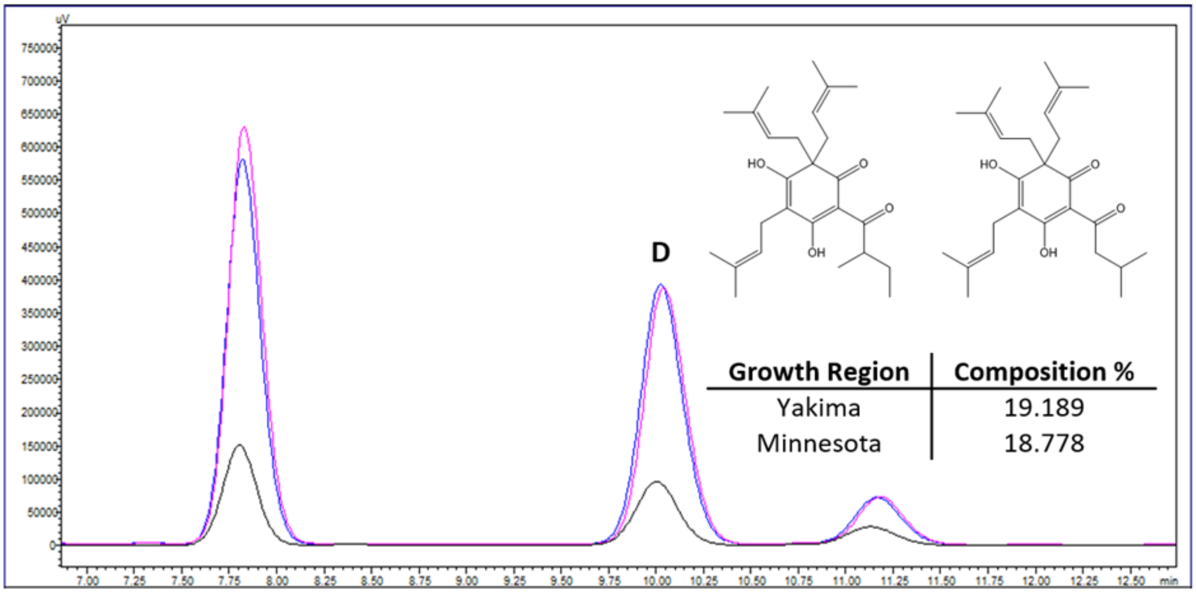

Figure S4. Overlayed HPLC-DAD chromatograms of the ASBC ICE-4 standard (black), Washington hops (blue), and Minnesota hops (pink). The structure of adlupulone (left) and lupulone (right) are shown as an inset in the upper right with a table corresponding to the composition values in the lower right.

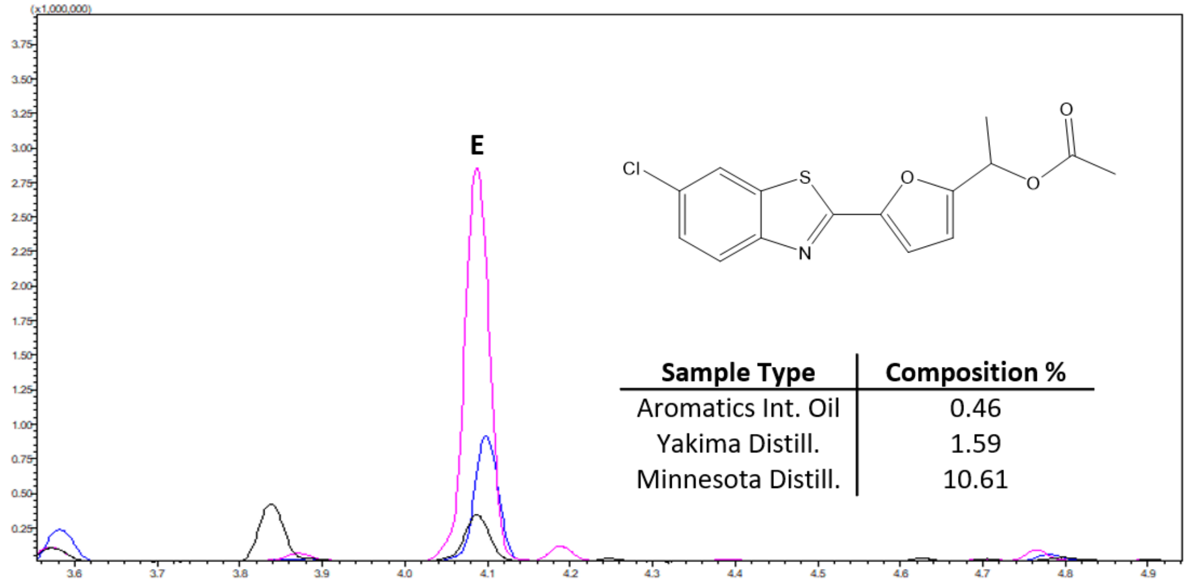

Figure S5. HS-GC-MS chromatogram comparison of Aromatics International Cascade Hop Oil (black), Washington hop distillate (blue), and Minnesota hop distillate (pink). The structure of 1-(5-(6-Chlorobenzo[d]thiazol-2-yl)furan-2-yl)ethyl acetate is shown as an inset in the upper right and a table of the preliminary relative composition values are provided as a table in the lower right.

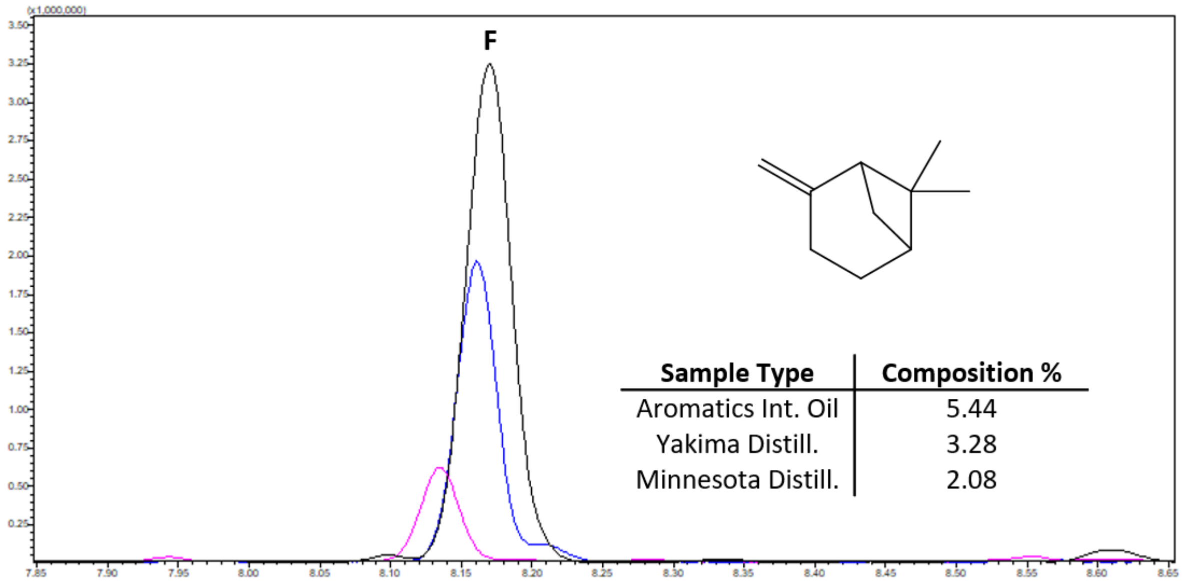

Figure S6. HS-GC-MS chromatogram comparison of Aromatics International Cascade Hop Oil (black), Washington hop distillate (blue), and Minnesota hop distillate (pink). The structure of β-pinene is shown as an inset in the upper right and a table of the preliminary relative composition values are provided as a table in the lower right.

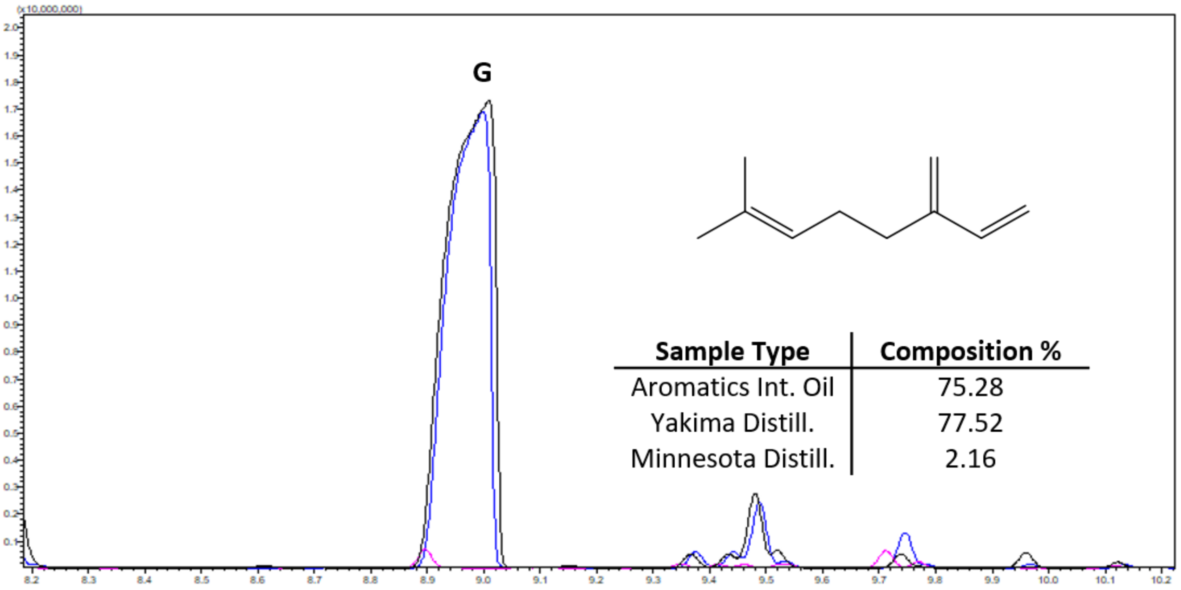

Figure S7. HS-GC-MS chromatogram comparison of Aromatics International Cascade Hop Oil (black), Washington hop distillate (blue), and Minnesota hop distillate (pink). The structure of β-myrcene is shown as an inset in the upper right and a table of the preliminary relative composition values are provided as a table in the lower right.

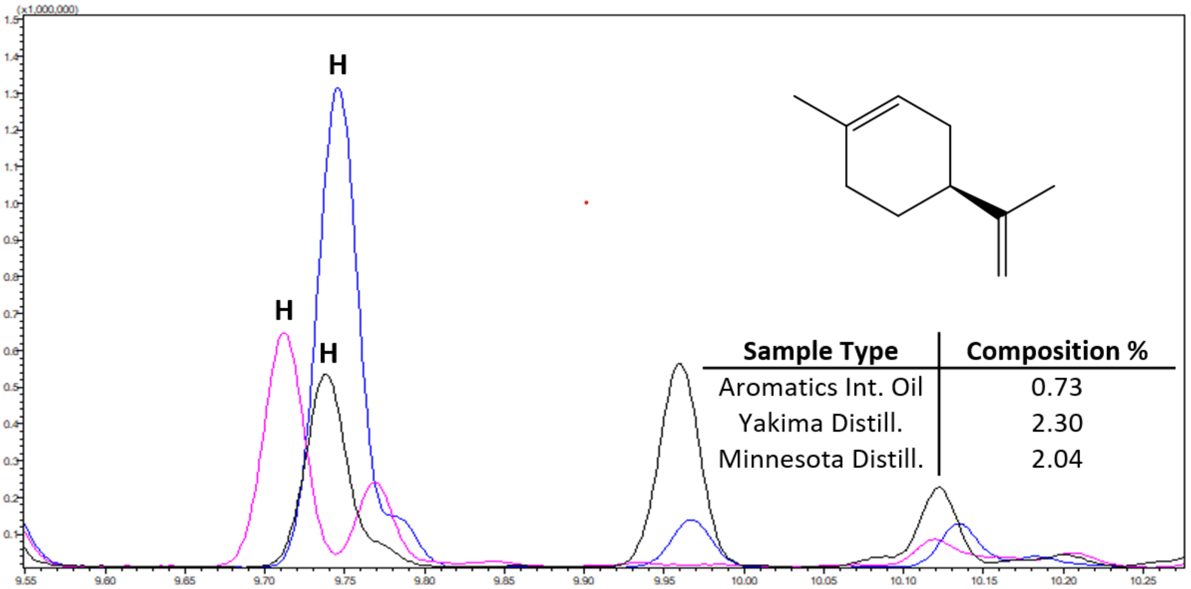

Figure S8. HS-GC-MS chromatogram comparison of Aromatics International Cascade Hop Oil (black), Washington hop distillate (blue), and Minnesota hop distillate (pink). The structure of D-limonene is shown as an inset in the upper right and a table of the preliminary relative composition values are provided as a table in the lower right.

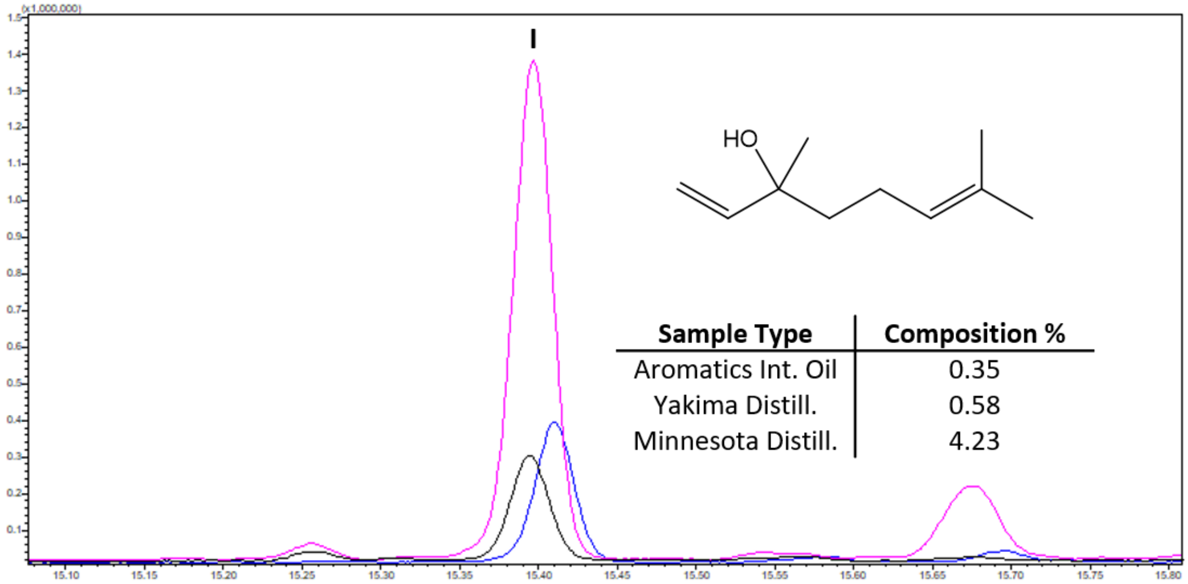

Figure S9. HS-GC-MS chromatogram comparison of Aromatics International Cascade Hop Oil (black), Washington hop distillate (blue), and Minnesota hop distillate (pink). The structure of linalool is shown as an inset in the upper right and a table of the preliminary relative composition values are provided as a table in the lower right.

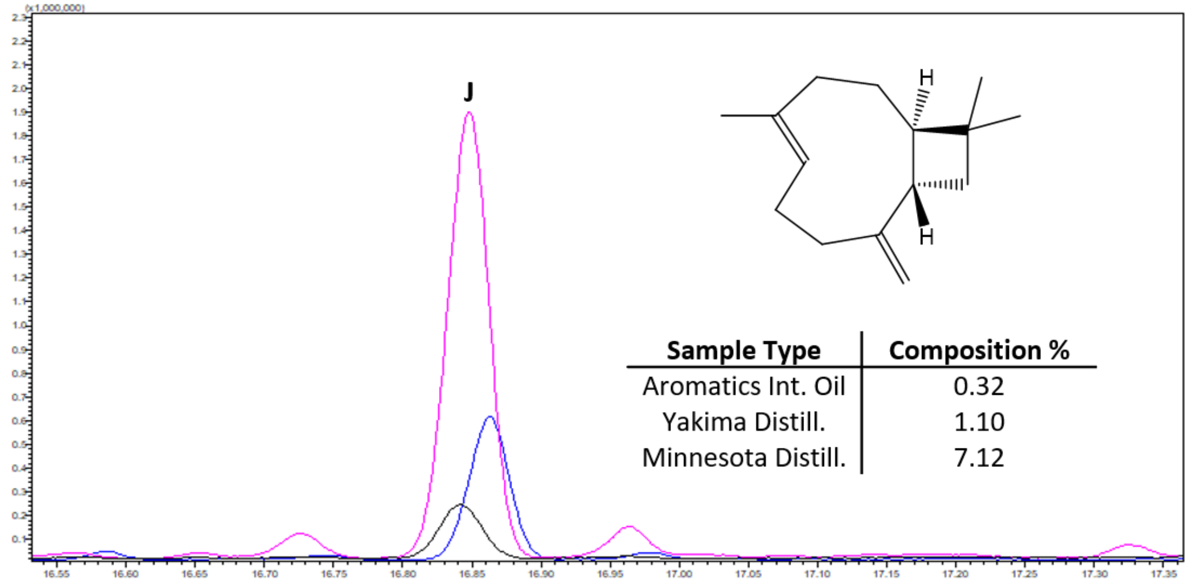

Figure S10. HS-GC-MS chromatogram comparison of Aromatics International Cascade Hop Oil (black), Washington hop distillate (blue), and Minnesota hop distillate (pink). The structure of caryophyllene (β-carophyllene) is shown as an inset in the upper right and a table of the preliminary relative composition values are provided as a table in the lower right.

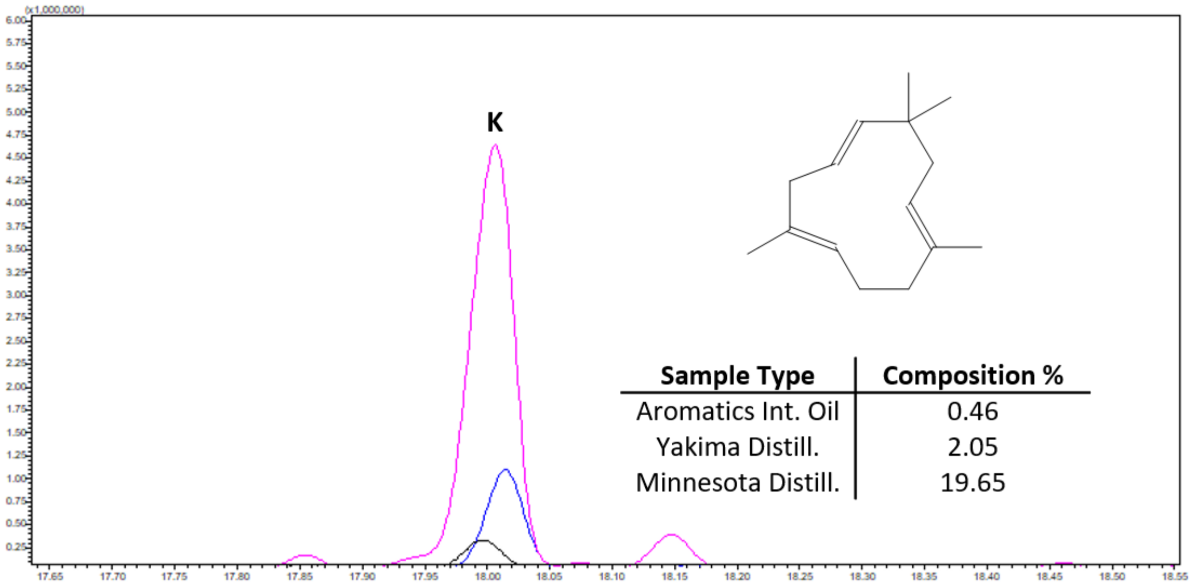

Figure S11. HS-GC-MS chromatogram comparison of Aromatics International Cascade Hop Oil (black), Washington hop distillate (blue), and Minnesota hop distillate (pink). The structure of humulene (α-caryophyllene) is shown as an inset in the upper right and a table of the preliminary relative composition values are provided as a table in the lower right.

Supplemental Table 1. The Aromatics International Cascade Hops Oil HS-GC-MS chromatogram data values are reported as well as the qualification of the peaks, and the corresponding CAS names with respective odor descriptions if available. The CAS names highlighted in yellow indicate compounds that were present in all three sample types. The CAS names highlighted in orange indicate compounds that were also present in the GC-MS chromatogram for the Yakima Cascade distillate. The Area % highlighted in green indicates the top five compounds with the greatest area percentage or relative composition percentage. The SI values highlighted in grey indicate SI values below 90.

Aromatics International Cascade Oil							
**Peak #**	**Ret. Time**	**Area %**	**SI**	**CAS #**	**Compound Name**	**CAS Name**	**Odor Description**
1	3.839	0.61	90	0 - 00 - 0	Oxalic acid, isohexyl neopentyl ester	-	-
2	4.088	0.46	90	0 - 00 - 0	1-(5-(6-Chlorobenzo[d]thiazol-2-yl)furan-2-yl)ethyl acetate	-	-
3	5.200	0.33	0	0-0-0		-	-
4	6.676	0.99	97	7785 - 70 - 8	(1R)-2,6,6-Trimethylbicyclo[3.1.1]hept-2-ene	D-(+)-alpha-pinene	harsh terpene aromatic minty
5	7.665	3.62	97	97 - 85 - 8	Propanoic acid, 2-methyl-, 2-methylpropyl ester	isobutyl isobutyrate	ethereal fruity tropical fruit pineapple grape skin banana
6	8.171	5.44	96	127 - 91 - 3	2(10)-Pinene	beta-pinene	cooling, woody, piney and turpentine-like with a fresh minty, eucalyptus and camphoraceous note with a spicy peppery and nutmeg nuance
7	9.009	75.28	97	123 - 35 - 3	.beta.-Myrcene	beta myrcene	peppery terpene spicy balsam plastic Terpy, herbaceous, woody with a rosy celery and carrot nuance
8	9.367	0.69	95	54004 - 43 - 2	2-Pentanol, propanoate	-	-
9	9.434	0.65	95	2050-01-3	Propanoic acid, 2-methyl-, 3-methylbutyl ester	isoamyl isobutyrate or isopentyl isobutyrate	fruity ethereal tropical green grape cherry unripe banana apple cocoa Sweet, fruity, estry and green with a waxy nuance
10	9.481	3.84	97	2445 - 69 - 4	Propanoic acid, 2-methyl-, 2-methylbutyl ester	2-methyl butyl isobutyrate	fruity ethereal tropical banana
11	9.523	0.74	86	129692 - 96 - 2	4-Butoxy-5,7-dinitro-benzo[1,2,5]thiadiazole	-	-
12	9.739	0.73	95	5989 - 27 - 5	D-Limonene	d-limonene	citric, lively, nuances of tangerine, celery and lemon oil
13	9.960	0.72	96	555 - 10 - 2	Cyclohexene, 3-methylene-6-(1-methylethyl)-	beta-phellandrene	mint terpentine
14	10.123	0.22	89	72101 - 07 - 6	Diphosphoric acid, diisooctyl ester	-	-
15	10.310	0.45	94	2177 - 83 - 5	Hexanoic acid, 5-methyl-, methyl ester	methyl 5-methyl hexanoate	not for fragrance
16	10.506	0.24	95	3338 - 55 - 4	1,3,6-Octatriene, 3,7-dimethyl-, (Z)-	(Z)-beta-ocimene	warm floral herb flower sweet
17	11.134	0.48	96	106 - 73 - 0	Heptanoic acid, methyl ester	methyl heptanoate	sweet, fruity and green, with a waxy apple like note
18	12.072	0.56	83	2519 - 37 - 1	Methyl 6-methyl heptanoate	methyl 6-methyl heptanoate	not for fragrance
19	12.107	0.54	82	5405 - 58 - 3	Hexane, 1,1'-[ethylidenebis(oxy)]bis-	acetaldehyde dihexyl acetal	sweet green ethereal waxy herbal
20	12.748	1.17	97	928 - 96 - 1	3-Hexen-1-ol, (Z)-	(Z)-3-hexen-1-ol	fresh green cut grass foliage vegetable herbal oily Green, grassy, melon rind-like with a pungent freshness
21	13.077	1.11	97	928 - 95 - 0	2-Hexen-1-ol, (E)-	(E)-2-hexen-1-ol	fresh green leafy fruity unripe banana Fresh fatty green, fruity, vegetative, with leafy and herbal nuances
22	15.395	0.35	97	78 - 70 - 6	Linalool	linalool	citrus floral sweet bois de rose woody green blueberry Citrus, orange, floral, terpy, waxy and rose
23	16.842	0.32	90	87 - 44 - 5	Caryophyllene	beta-caryophyllene	sweet woody spice clove dry
24	17.997	0.46	97	6753 - 98 - 6	alpha.-Humulene	humulene or alpha caryophyllene	woody, oceanic water, spicy clove

Supplemental Table 2. The Yakima Cascade distillate GC-MS chromatogram data values are reported as well as the qualification of the peaks, and the corresponding CAS names with respective odor descriptions if available. The CAS names highlighted in yellow indicate compounds that were present in all three sample types. The CAS names highlighted in blue indicate compounds that were also present in the GC-MS chromatogram for the Minnesota Cascade distillate. The CAS names highlighted in orange indicate compounds that were also present in the GC-MS chromatogram for the Aromatics International Cascade Hops Oil. The Area % highlighted in green indicates the top five compounds with the greatest area percentage or relative composition percentage. The SI values highlighted in grey indicate SI values below 90.

Yakima Cascade Distillate							
**Peak #**	**Ret. Time**	**Area %**	**SI**	**CAS #**	**Compound Name**	**CAS Name**	**Odor**
1	3.583	0.44	97	13292 - 87 - 0	Borane-methyl sulfide complex	-	sulfury onion sweet corn vegetable cabbage tomato green radish Sulfurous, dimethyl sulfide, creamy, tomato, fishy, scallop, berry fruity and vegetative nuances
2	4.099	1.59	89	0 - 00 - 0	1-(5-(6-Chlorobenzo[d]thiazol-2-yl)furan-2-yl)ethyl acetate	-	-
3	5.212	0.30	85	5412 - 92 - 0	2-Octanamine, N-(1-methylheptyl)-	-	-
4	6.436	0.31	-	-	-	-	-
5	6.681	0.56	91	7785 - 70 - 8	(1R)-2,6,6-Trimethylbicyclo[3.1.1]hept-2-ene	D-(+)-alpha-pinene	harsh terpene aromatic minty
6	6.725	1.35	84	110990 - 15 - 3	Propanamide, 2-methyl-2-(1-oxobutoxy)-N-(3-trifluoromethyl-4-nitrophenyl)-	-	-
7	7.661	0.68	82	0 - 00 - 0	Oxalic acid, neopentyl pentyl ester	-	-
8	8.162	3.28	95	127 - 91 - 3	2(10)-Pinene	beta-pinene	cooling, woody, piney and turpentine-like with a fresh minty, eucalyptus and camphoraceous note with a spicy peppery and nutmeg nuance
9	8.999	77.52	96	123 - 35 - 3	.beta.-Myrcene	beta myrcene	peppery terpene spicy balsam plastic Terpy, herbaceous, woody with a rosy celery and carrot nuance
10	9.376	0.97	85	129967 - 99 - 3	d-Xylitol, 1,3,5-trideoxy-3-nitro-, 4-(2,2-dimethylpropanoate)	-	-
11	9.442	0.99	85	0 - 00 - 0	Diglycolic acid, di(3-methylbutyl) ester	-	wine waxy oily fatty peach Winey, alcoholic, fatty, creamy, yeasty and fusel
12	9.489	4.03	98	2445 - 69 - 4	Propanoic acid, 2-methyl-, 2-methylbutyl ester	2-methyl butyl isobutyrate	fruity ethereal tropical banana
13	9.54	0.38	80	129692 - 96 - 2	4-Butoxy-5,7-dinitro-benzo[1,2,5]thiadiazole	-	-
14	9.746	2.30	96	5989 - 27 - 5	D-Limonene	d-limonene	citric, lively, nuances of tangerine, celery and lemon oil
15	13.538	1.21	95	539 - 52 - 6	Furan, 3-(4-methyl-3-pentenyl)-	perillene	woody
16	15.411	0.58	97	78 - 70 - 6	Linalool	linalool	citrus floral sweet bois de rose woody green blueberry, citrus, orange, floral, terpy, waxy and rose
17	16.864	1.10	97	87 - 44 - 5	Caryophyllene	beta-caryophyllene	sweet woody spice clove dry
18	17.402	0.35	95	18794 - 84 - 8	(E)-.beta.-Farnesene	(E)-beta-farnesene	woody citrus herbal sweet
19	18.015	2.05	97	6753 - 98 - 6	.alpha.-Humulene	humulene or alpha caryophyllene	woody, oceanic water, spicy clove

Supplemental Table 3. The Minnesota Cascade distillate GC-MS chromatogram data values are reported as well as the qualification of the peaks, and the corresponding CAS names with respective odor descriptions if available. The CAS names highlighted in yellow indicate compounds that were present in all three sample types. The CAS names highlighted in blue indicate compounds that were also present in the GC-MS chromatogram for the Yakima Cascade distillate. The Area % highlighted in green indicates the top five compounds with the greatest area percentage or relative composition percentage. The SI values highlighted in grey indicate SI values below 90.

Minnesota Cascade Distillate							
Peak #	Ret. Time	Area %	SI	CAS #	Compound Name	CAS Name	Odor Description
1	3.263	2.89	86	0 - 00 - 0	21-(2,4,10-trioxa-adamant-3-yl)-heneicosan-3,5,11,13,15,17,19-heptaen-7,9-diyn-2-one	-	-
2	4.088	10.61	88	0 - 00 - 0	1-(5-(6-Chlorobenzo[d]thiazol-2-yl)furan-2-yl)ethyl acetate	-	-
3	5.152	0.95	-	-	-	-	-
4	5.202	1.75	85	5412 - 92 - 0	2-Octanamine, N-(1-methylheptyl)-	-	-
5	5.365	1.25	87	94514 - 30 - 4	(1,1-dimethyl-3-oxo-butyl)-[(1,1-dimethyl-3-oxo-butyl)-oxido-amino]-oxo-ammonium	-	-
6	6.421	2.60	95	108 - 10 - 1	Methyl Isobutyl Ketone	isobutyl methyl ketone	sharp solvent green herbal fruity dairy spice Sharp solvent-like with green, herbal, fruity and dairy nuances
7	6.547	0.64	96	556 - 24 - 1	Methyl isovalerate	methyl isovalerate	strong apple fruity pineapple Diffusive, sweet estery, fruity with tutti frutti nuances
8	6.606	0.50	-	-	-	-	-
9	6.713	15.98	98	115 - 18 - 4	3-Buten-2-ol, 2-methyl-	2-methyl-3-buten-2-ol	herbal earthy oily
10	7.634	1.02	95	66 - 25 - 1	Hexanal	hexanal (aldehyde C-6)	fresh green fatty aldehydic grass leafy fruity sweaty green, fatty, leafy, vegetative, fruity and clean with a woody nuance
11	8.136	2.08	96	127 - 91 - 3	2(10)-Pinene	beta-pinene	cooling, woody, piney and turpentine-like with a fresh minty, eucalyptus and camphoraceous note with a spicy peppery and nutmeg nuance
12	8.896	2.16	95	123 - 35 - 3	.beta.-Myrcene	beta myrcene	peppery terpene spicy balsam plastic Terpy, herbaceous, woody with a rosy celery and carrot nuance
13	9.713	2.04	96	5989 - 27 - 5	D-Limonene	d-limonene	citric, lively, nuances of tangerine, celery and lemon oil
14	9.769	0.62	84	0 - 00 - 0	1-(1,1-Dioxo-tetrahydro-1.lambda.(6)-thiophene-3-sulfonyl)-piperidine	-	-
15	12.07	1.03	88	2445 - 71 - 8	2-Hexenoic acid, 4-methyl-, methyl ester	-	-
16	12.112	1.20	95	110 - 93 - 0	5-Hepten-2-one, 6-methyl-	methyl heptenone	citrus green musty lemongrass apple Fruity, apple, musty, ketonic and creamy with slight cheesy and banana nuances
17	12.868	1.47	95	344294 - 72 - 0	4H-Cyclopenta[c]furan, hexahydro-1,1-dimethyl-4-methylene-	hop ether	not for fragrance
18	13.085	1.70	85	4727 - 83 - 7	(2,5-dimethyl-6-methylol-cyclohex-3-en-1-yl)methanol	-	-
19	13.522	3.73	95	539 - 52 - 6	Furan, 3-(4-methyl-3-pentenyl)-	perillene	woody
20	14.999	0.90	95	3856 - 25 - 5	Copaene	alpha-copaene or copaene	woody spicy honey
21	15.397	4.23	97	78 - 70 - 6	Linalool	linalool	citrus floral sweet bois de rose woody green blueberry Citrus, orange, floral, terpy, waxy and rose
22	16.216	1.95	91	5392 - 40 - 5	(2E)-3,7-Dimethyl-2,6-octadienal	citral	sharp lemon sweet Fresh, juicy, lemon peel, with a sweet tangy green nuance
23	16.37	0.81	96	13474 - 59 - 4	trans-.alpha.-Bergamotene	(E)-alpha-bergamotene	woody warm tea
24	16.464	1.96	97	112 - 12 - 9	2-Undecanone or nonyl methyl ketone	2-undecanone	waxy fruity creamy fatty orris floral Waxy, fruity, ketonic with fatty pineapple nuances
25	16.849	7.12	96	87 - 44 - 5	Caryophyllene	beta-caryophyllene	sweet woody spice clove dry
26	18.007	19.65	96	6753 - 98 - 6	.alpha.-Humulene	humulene or alpha caryophyllene	woody, oceanic water, spicy clove
27	18.148	1.35	96	30021 - 74 - 0	.gamma.-Muurolene	gamma-muurolene	herbal woody spice
28	18.789	4.52	97	17066 - 67 - 0	Naphthalene, decahydro-4a-methyl-1-methylene-7-(1-methylethenyl)-, [4aR-(4a.alpha.,7.alpha.,8a.beta.)]-	beta-selinene	herbal
29	19.26	0.79	96	39029 - 41 - 9	Naphthalene, 1,2,3,4,4a,5,6,8a-octahydro-7-methyl-4-methylene-1-(1-methylethyl)-, (1.alpha.,4a.beta.,8a.alpha.)-	(R)-gamma-cadinene	herbal woody
30	20.021	0.79	96	106 - 24 - 1	Geraniol or trans-3,7-dimethyl-2,7-octadien-1-ol	geraniol	sweet floral fruity rose waxy citrus floral, sweet, rosey, fruity and citronella-like with a citrus nuance
31	24.754	1.72	96	19888 - 34 - 7	(1R,3E,7E,11R)-1,5,5,8-Tetramethyl-12-oxabicyclo[9.1.0]dodeca-3,7-diene	humulene oxide II	-
